# Association of family income to poverty ratio and vibration-controlled transient elastography quantified degree of hepatic steatosis in U.S. adolescents

**DOI:** 10.3389/fendo.2023.1160625

**Published:** 2023-03-23

**Authors:** Meiling Tang, Mingjiang Liu, Ya Zhang, Ruijie Xie

**Affiliations:** ^1^ Department of Pediatrics, University of South China Affiliated Nanhua Hospital, Hengyang, China; ^2^ Department of Microsurgery, University of South China. Hengyang Affiliated Nanhua Hospital, Hengyang, China; ^3^ Department of Gland Surgery, University of South China. Hengyang Affiliated Nanhua Hospital, Hengyang, China

**Keywords:** ratio of family income to poverty, hepatic steatosis, NAFLD, NHANES, socioeconomic status

## Abstract

**Introduction:**

Inequality in socioeconomic status plays an important role in the prevalence of metabolic diseases in adolescents. The purpose of this study was to explore the association between family income and the degree of hepatic steatosis quantified by vibration-controlled transient elastography (VCTE) among U.S. adolescents.

**Methods:**

This cross-sectional study included two cycles of the National Health and Nutrition Examination Survey (NHANES) 2017-2020. Multivariate linear regression and smoothing curve fitting were used to investigate the linear and nonlinear relationship between PIR and hepatic steatosis, respectively. Subgroup analysis and interaction tests were used to test whether this relationship was stable across groups.

**Results:**

Of the 1,574 adolescent participants, 456 lived in poor households and 307 lived in wealthy households. After adjusting for all covariates, PIR (Ratio of family income to poverty) was significantly negatively associated with the degree of hepatic steatosis [-4.78 (-7.39, -2.17)], and this remained stable after converting PIR to a categorical variable. In addition, this significant negative association was more pronounced in women [-7.62 (-11.38, -3.87)], non-Hispanic blacks [-7.19 (-14.43, 0.06)], Mexican Americans [-6.80 (-13.63, 0.03)], and participants with BMI >30 cm^2^ [-10.83 (-19.70, -1.96)].

**Conclusions:**

PIR was significantly and negatively associated with the degree of hepatic steatosis in US adolescents. Additional prospective studies are needed to confirm our findings.

## 1 Introduction

Over the past three decades, non-alcoholic fatty liver disease (NAFLD) has developed into the most common cause of chronic liver disease worldwide (1, 2), with an alarming 36.1% prevalence of NAFLD in children and adolescents in the context of obesity (3). Worryingly, there is epidemiological evidence that this number will continue to rise in the future (4). The persistence of NAFLD in childhood into adulthood may be a cause of serious liver and metabolic disease and is critical for early risk factor detection and screening (5).

Many biomarkers have been shown in epidemiological studies to be strongly associated with NAFLD in the past (6–8). However, non-negligible sociological factors are also receiving increasing attention in the liver metabolism of children and adolescents (9). Metabolic disorders are now increasingly common in young adults, exhibit gender and racial differences, and are attributed to many interrelated factors such as genetic, environmental, and social factors. Socioeconomic disadvantage is common among U.S. adolescents, so studying the impact of family SES on the emergence of metabolic disease in early adolescence may help to prevent and manage the social actions and policies of health and economic burden throughout the life course (10, 11). A scoping review that included seven studies from different countries and regions showed a significant increase in obesity rates among adolescents with low socioeconomic status (SES) (12, 13). In addition, parental income status has been shown to be negatively associated with the prevalence of metabolic syndrome in adolescents (14, 15). Unequal socioeconomic status can affect normal organ metabolism in adolescents in terms of nutritional intake (16, 17), lifestyle habits (18), and metal exposure (19). However, there is no evidence to suggest whether adolescent household income is associated with the degree of hepatic steatosis as opposed to the obvious physical characteristics of appearance.

Therefore, we performed a cross-sectional study based on National Health and Nutrition Examination Survey (NHANES) 2017-2020 to investigate the relationship between family income to poverty ratio and the degree of hepatic steatosis quantified by vibration-controlled transient elastography (VCTE) among US adolescents.

## 2 Methods

### 2.1 Study population

**Figure 1:**
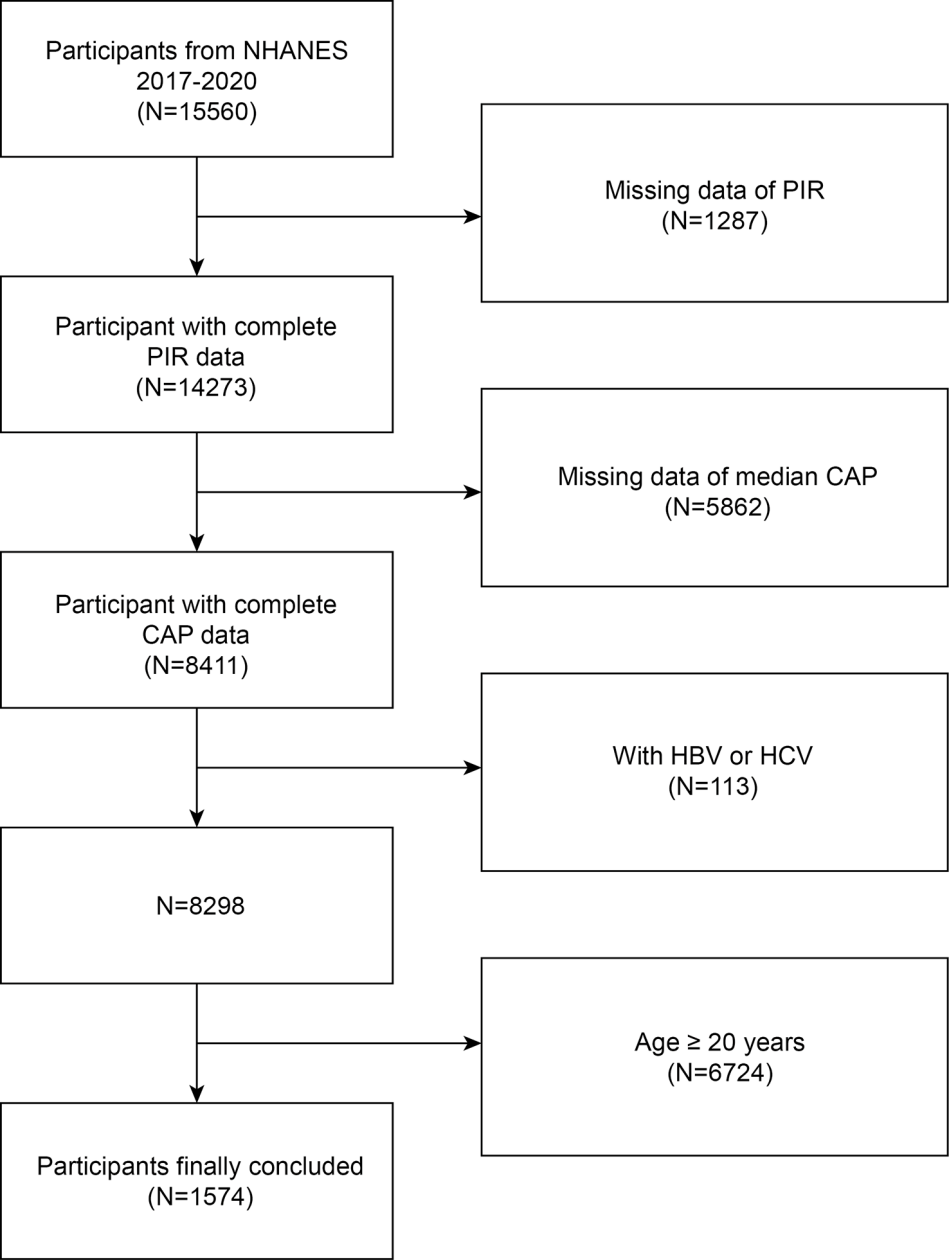
Flow chart of participants selection. NHANES, National Health and Nutrition Examination Survey; CAP, Controlled Attenuation Parameter; HBV, Hepatitis B; HCV, Hepatitis C; Ratio of family income to poverty, PIR.

The National Center for Health Statistics at the Centers for Disease Control and Prevention gathered data for the NHANES 2017-2020, which we examined. For proper coverage of the noninstitutionalized civilian population of the country, this cross-sectional survey employs stratified multistage probability cluster sampling (20–22). The National Center for Health Statistics (NCHS) Research Ethics Review Board authorized the study protocol. At the time of recruiting, all subjects provided written consent. We excluded 1287 participants without PIR data, 5862 participants without CAP data, 113 participants with Hepatitis B or C, and 6724 age more than 20 years. The study eventually included 1574 adolescent participants (**Figure 1**).

### 2.2 Study variables

We used the poverty-to-income ratio (PIR), an index of income related to household needs, by calculating annual changes in household size and cost of living and tracking the consumer price index from household income and federally determined poverty thresholds (23, 24). We divide the PIR into three levels, low-income level (PIR < 1), middle-income level (PIR 1-4), and high-income level (PIR > 4) (25).

The degree of hepatic steatosis was assessed using controlled attenuation data obtained by VCTE (26). Measurements were obtained by a professional operator from each participant for at least 10 measurements, and the device calculated a median controlled attenuation parameter (CAP) with a CAP value ranging from 100-400 dB/m, with higher values indicating higher liver fat content. A recent study describes the cut-off values for the grade of steatosis (27).

Age, gender, race, diabetes status, BMI, waist circumference, physical activities, triglycerides, high-density lipoprotein cholesterol (HDL-C) and low-density lipoprotein cholesterol (LDL-C) were all covariates in this study. The interpretation, measurement and calculation of all variables can be found on the official NHANES website (https://www.cdc.gov/nchs/nhanes/).

### 2.3 Statistical analysis

All analyses were performed with R (version 4.2) and Empowerstats (version 4.1). The chi-square test and t-test were used to assess the demographic characteristics of the participants by PIR classifications. Multivariate linear regression analyses were used to investigate the associations between PIR and CAP. The nonlinear association between PIR and CAP was explored by weighted generalized additive model and smoothed curve fitting. The parallel mediation model uses individual indicators as mediators. Subgroup analysis and interaction tests were used to investigate the relationship between PIR and CAP in different groups. A two-tailed P value < 0.05 was considered statistically significant.

## 3 Results

### 3.1 Baseline characteristics

The weighted characteristics were separated into three categories (low income, middle income, and high income) based on PIR. In total, 822 male and 752 female adolescents were involved, the mean PIR among all participants was 2.17. Notably, more than 28.9% of adolescents live in households with incomes below the poverty line (PIR<1), compared to 19.5% of adolescents living in wealthy households. Adolescents living below the poverty line are more likely to be of a race other than non-Hispanic white and have higher BMI, waist circumference and HDL-C than adolescents living in wealthy households (**Table 1**).

**Table 1:** Basic characteristics of participants by family PIR.

Characteristics	Low income (PIR < 1, N=456)	Middle income (PIR 1-4, N=811)	High income (PIR ≥ 4, N=307)	*P*-value
Age (years)	15.60 ± 2.34	15.15 ± 2.20	15.50 ± 2.15	0.001
Sex, n (%)				0.089
Male	231 (50.66%)	444 (54.75%)	147 (47.88%)	
Female	225 (49.34%)	367 (45.25%)	160 (52.12%)	
Race/ethnicity, n (%)				<0.001
Non-Hispanic White	113 (24.78%)	269 (33.17%)	147 (47.88%)	
Non-Hispanic Black	154 (33.77%)	198 (24.41%)	33 (10.75%)	
Mexican American	68 (14.91%)	141 (17.39%)	28 (9.12%)	
Other race/multiracial	121 (26.54%)	203 (25.03%)	99 (32.25%)	
Diabetes, n (%)				0.409
Yes	2 (0.44%)	7 (0.86%)	0 (0.00%)	
No	448 (98.25%)	796 (98.15%)	306 (99.67%)	
Borderline	6 (1.32%)	8 (0.99%)	1 (0.33%)	
Days of physical activities, n (%)				0.060
0-3	157 (34.45%)	288 (30.53%)	89 (28.87%)	
4-7	299 (65.55%)	523 (69.47%)	218 (71.13%)	
BMI (kg/m^2^)	25.49 ± 7.13	25.05 ± 7.14	23.48 ± 5.38	<0.001
< 25 25-30 >30	259 (57.43%) 94 (20.84%) 98 (21.73%)	487 (60.42%) 154 (19.11%) 165 (20.47%)	212 (69.51%) 62 (20.33%) 31 (10.16%)	
Triglycerides (mg./L) HDL-C (mg/L) LDL-C (mg/L)	72.04 ± 43.60 50.37 ± 11.27 89.46 ± 26.94	68.28 ± 39.86 51.88 ± 12.39 85.87 ± 24.57	69.88 ± 30.51 52.56 ± 11.45 89.22 ± 25.85	0.595 0.040 0.237
Waist circumference (cm)	84.49 ± 17.31	83.88 ± 16.71	80.38 ± 13.06	0.002
Median CAP (dB/m)	227.27 ± 56.45	223.58 ± 54.16	210.83 ± 48.88	<0.001

Mean ± SD for continuous variables: the P value was calculated by the weighted linear regression model.

(%) for categorical variables: the P value was calculated by the weighted chi-square test.

PIR, Ratio of family income to poverty; BMI, body mass index; HDL-C, High-density lipoprotein cholesterol; LDL-C, low-density lipoprotein cholesterol; CAP, Controlled Attenuation Parameter.

### 3.2 Association between PIR and hepatic steatosis

**Figure 2:**
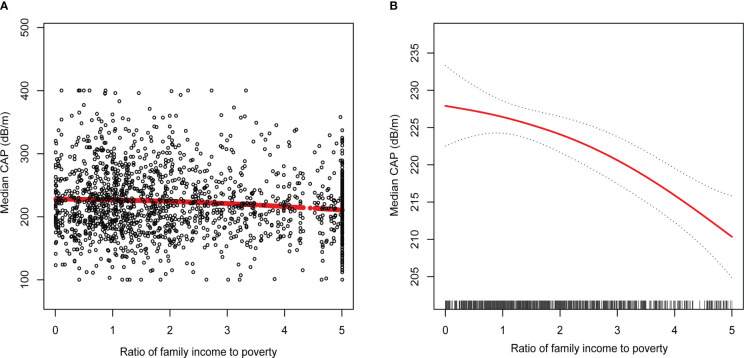
The association between PIR and degree of hepatic steatosis. **(A)** Each black point represents a sample. **(B)** The solid red line represents the smooth curve fit between variables. Blue bands represent the 95% of confidence interval from the fit. Controlled Attenuation Parameter; Ratio of family income to poverty, PIR.

**Table 2** shows the results of multivariate linear regression analyses for three models. There was a significant negative linear association between PIR and CAP in unadjusted model [-3.51 (-5.17, -1.84)]. The negative correlation between PIR and CAP remains significant even after adjusting for all covariates [-4.78 (-7.39, -2.17)]. We further investigated the association between different PIR levels and CAP after transforming PIR into categorical variables. In the fully adjusted model, the significant negative association between different levels of PIR and CAP persisted. Using the PIR of low-income participants as the reference group, the median CAP significantly decreased by 7.73 dB/m for every 1-score increase in PIR of participants in the middle-income group [-7.73 (-17.18, 1.73)] and by 21.85 dB/m for every 1-score increase in PIR of participants in the high-income group [-21.85 (-33.94, -9.76)]. In addition, we further performed generalized model smoothed curve fitting to confirm the non-linear relationship between PIR and CAP. The results validated a negative non-linear negative relationship between PIR and CAP (**Figure 2**).

**Table 2:** The associations between family PIR and hepatic steatosis.

Exposure	Model 1 [β (95% CI)]	Model 2 [β (95% CI)]	Model 3 [β (95% CI)]
Ratio of family income to poverty	-3.51 (-5.17, -1.84)	-3.65 (-5.37, -1.93)	-4.78 (-7.39, -2.17)
PIR classification			
Low income (PIR < 1)	ref	ref	ref
Middle income (PIR 1-4)	-3.69 (-9.87, 2.49)	-4.40 (-10.63, 1.83)	-7.73 (-17.18, 1.73)
High income (PIR ≥ 4)	-16.44 (-24.23, -8.64)	-16.89 (-24.85, -8.93)	-21.85 (-33.94, -9.76)

Model 1: no covariates were adjusted. Model 2: age, gender, and race were adjusted. Model 3: age, gender, race, BMI, physical activities, diabetes status, Triglycerides, HDL-C and LDL-C were adjusted.

PIR, Ratio of family income to poverty; BMI, body mass index; HDL-C, High-density lipoprotein cholesterol; LDL-C, low-density lipoprotein cholesterol; CAP, Controlled Attenuation Parameter.

### 3.3 Subgroup analyses

In subgroup analyses stratified by sex, race, age, and BMI, we found inconsistent associations between PIR and CAP (**Table 3**). Although the association between PIR and CAP remained negative in all subgroups, this linear negative association remained significant only in women, in participants aged 12-15 years, and in those with a BMI greater than or equal to 30. More importantly, the results of the interaction test showed that sex modified the association between PIR and CAP among adolescents, and no significant dependence of race, age, and BMI on this negative correlation.

**Table 3:** Subgroup analysis of the association between family PIR and median CAP.

Subgroup	OR (95%CI)	P for interaction
Sex		0.020
Male	-1.92 (-5.61, 1.78)	
Female	-7.62 (-11.38, -3.87)	
Age		0.820
12-15	-6.28 (-9.85, -2.70)	
16-19	-3.36 (-6.98, 0.27)	
Race/ethnicity		0.867
Non-Hispanic White	-1.96 (-5.93, 2.02)	
Non-Hispanic Black	-7.19 (-14.43, 0.06)	
Mexican American	-6.80 (-13.63, 0.03)	
Other race/multiracial	-2.17 (-5.19, 0.86)	
BMI		0.065
<24.9	-1.03 (-3.58, 1.53)	
25-29.9	-2.35 (-7.50, 2.80)	
≥30	-10.83 (-19.70, -1.96)	

Age, gender, race, BMI, physical activities, diabetes status, Triglycerides, HDL-C and LDL-C were adjusted. In the subgroup analyses, the model is not adjusted for the stratification variable itself.

PIR, Ratio of family income to poverty; BMI, body mass index; CAP, Controlled Attenuation Parameter.

## 4 Discussion

In this study, we assessed the association between PIR and degree of hepatic steatosis in US adolescents and found that low PIR was significantly associated with higher degree of hepatic steatosis, and this association was more prominent in females, non-Hispanic black, Mexican American, and BMI >30 kg/m^2^ participants. To our knowledge, this is the first epidemiological study to investigate PIR and degree of hepatic steatosis in an adolescent population.

Previous epidemiological studies have highlighted that SES inequality is closely related to metabolic syndrome and that this association varies across regions and countries (28, 29). A cohort study from Sweden used parental physical labor at age 16 as a criterion for low SES and showed that disadvantaged socioeconomic status in childhood or adolescence specifically increased the risk of metabolic syndrome in women (30). In addition, two studies, also from NHANES 1999-2002, demonstrated that the higher the PIR and education level, the lower the probability of obesity, hypertension and hyperglycemia in adolescents (31, 32). Our results are consistent with the above findings, demonstrating that low PIR is significantly associated with higher BMI and waist circumference, and in addition, gender differences are not negligible in this association.

Evidence from the Western Australian Pregnancy Cohort Study, which included pregnancy-related characteristics of parents of 1170 17-year-old adolescents, demonstrated that lower family income at birth was significantly associated with NAFLD in male offspring when not coterminous with obesity (33). Although there is epidemiological evidence confirming a negative association between PIR and NAFLD in adolescents, these studies tend to diagnose NAFLD by liver enzyme and index calculations (34). Two main problems with such a diagnostic approach are that it does not accurately measure the severity of hepatic steatosis in participants and does not allow for a further description of the association between PIR and NAFLD (35). In addition, several studies have shown that the degree of hepatic steatosis calculated by liver enzymes or indices may be statistically biased and may significantly underestimate the number of participants with NAFLD in epidemiological studies (36). VCTE, as recommended by the American Gastroenterological Association for risk stratification and management of patients with NAFLD, is highly accurate in diagnosing the degree of hepatic steatosis and the degree of fibrosis (37, 38). Therefore, we used VCTE data to avoid diagnostic inaccuracies and the inability to quantify the degree of hepatic steatosis in the current study.

Explaining the PIR differences observed in adolescent hepatic steatosis is expected to be as complex as defining population differences in the metabolic syndrome because of the intricate interactions between the metabolic syndrome and multiple known disease factors, environmental factors, cultural factors, climatic factors, and genetic factors (39). However, through a review of studies describing the comorbidity of NAFLD in adolescents, understanding the prevalence and characteristics of hepatic steatosis in the adolescent population may provide potential evidence to help improve strategies to prevent NAFLD and related liver diseases in the adolescent population. Epidemiological evidence suggests that groups with higher household incomes adopt healthy but higher cost-of-living lifestyles early in life, while groups of children and adolescents in low-income households live with an increased prevalence of risky behaviors (40, 41). Based on observed forms including inability to afford healthy food, exposure to harmful environments, and lack of access to quality health services (42). In addition, ethnic and cultural differences in minority groups in high-income countries are also associated with a significantly higher risk of obesity and metabolic disease than other groups (43, 44). More importantly, the exposure of parents with low SES to non-communicable diseases (NCDs) during fetal life and infancy increases the risk of developing childhood NCDs (45). For example, offspring of diabetic parents exhibit earlier and more pronounced insulin resistance features in early adolescence, providing plausible evidence for biological factors (46). Recent epidemiological evidence also provides a novel explanation for the mechanism behind the negative association between PIR and hepatic steatosis: this negative association may derive mainly from mediating factors arising from PIR, such as diet quality and physical activity, with further effects of these variables on liver metabolism (47).

Our study has some limitations. First, due to the design of the cross-sectional study, we were unable to determine the causal relationship between PIR and degree of hepatic steatosis (48, 49). In addition, because the PIR is linked to a large number of variables, we were unable to include all covariates that had a potential impact on, which may lead to incomplete accuracy of the results. Despite these shortcomings, our study has several advantages. This study includes data from a large and representative cross-sectional survey. More importantly, this study confirms the association between PIR and hepatic steatosis and quantifies for the first time the effect of PIR on the degree of hepatic steatosis.

## 5 Conclusion

Our results suggest that PIR negatively correlated with degree of hepatic steatosis among U.S. adolescents. Differences of PIR in the population should be considered in the diagnosis and treatment of NAFLD.

## Data availability statement

Publicly available datasets were analyzed in this study. This data can be found here: https://www.cdc.gov/nchs/nhanes/.

## Ethics statement

The studies involving human participants were reviewed and approved by The National Center for Health Statistics (NCHS) Research Ethics Review Board. Written informed consent to participate in this study was provided by the participants’ legal guardian/next of kin.

## Author contributions

MT and RX designed the research. MT, ML, and YZ collected, analyzed the data, and drafted the manuscript. YZ and RX revised the manuscript. All authors contributed to the article and approved the submitted version.
